# Supplementary material for: A Real-World Comparison of the Safety Profile for Immune Checkpoint Inhibitors in Oncology Patients
Source: J Clin Med. 2025 Jan 9;14(2):388. doi: 10.3390/jcm14020388 (PMC11765622; doi:10.3390/jcm14020388)
Supplement: Supplementary file 1 [file jcm-14-00388-s001.zip › jcm-3393973-supplementary.pdf]

Major changes upon excluding durvalumab-treated patients and reanalyzing the data.

Table S1. Baseline clinical characteristics of patients treated with ICIs.

Characteristics	Nivolumab (n=221)	Pembrolizumab (n=126)	Atezolizumab (n=78)	P-value
Heart diseases				
Yes	15 (6.8)	12 (9.5)	6 (7.7)	0.657
No	206 (93.2)	114 (90.5)	72 (92.3)	
Labs [Median (IQR)]				
ALT (U/L)*	24.03 (7.9)	26.06 (9.6)	24.48 (5.8)	0.06

Table S2. Complications according to category and ICI medication.

Complication	Nivolumab (n=221)	Pembrolizumab (n=126)	Atezolizumab (n=78)	P-value
Thrombocytopenia				
Yes	2 (0.9)	7 (5.6)	2 (2.6)	0.032
No	219 (99.1)	119 (94.4)	72 (97.4)	
Gastrointestinal complication (colitis)				
Yes	10 (4.5)	2 (1.6)	1 (1.3)	0.187
No	211 (95.5)	124 (98.4)	77 (98.7)	

Table S3. Onset of complications and side effects according to ICI medication

Complication/side effect	Average Weeks elapsed until complication [Median (IQR)]	Weeks elapsed since treatment initiation until adverse effects occurrence [Median (IQR)]			
		Nivolumab (n=221)	Pembrolizumab (n=126)	Atezolizumab (n=78)	P-value
Cardiovascular	5.0 (NA)	NA	NA	5.0 (8.5)	NA
Gastrointestinal complications (colitis)	8.5 (19)	8.5 (18.5)	6.0 (38.0)	16.0 (0.0)	0.368

Table S4. Factors associated with complications and side effects of ICI medications.

Complication/side effect	Patients' factors		Coef (B)	S.E	Odds ratio	95% CI	P value
Hepatic complication	Gender	Male			Ref		
		Female	0.797	0.455	2.21	0.91 – 5.41	0.08
